# Educational Attainment and Gender Differences in Work–Life Balance for Couples across Europe: A Contextual Perspective

**DOI:** 10.17645/si.v8i4.2920

**Published:** 2020-10-09

**Authors:** Theocharis Kromydas

**Affiliations:** MRC/CSO Social and Public Health Sciences Unit, University of Glasgow, Glasgow, G3 7HR, UK

**Keywords:** division of labour, dual-earner households, gender inequalities, job quality, work–life balance

## Abstract

The current article aims to explain the interrelationships between the educational attainment of individuals living in house-holds with heterosexual partners, their work–life balance (WLB) and the macro-economic climate of the country they live in, using data from the European Social Survey. WLB is a complex concept, as it is not only determined by factors related to someone’s employment or domestic work and childcare responsibilities, but also by decisions informed by personal experiences and circumstances, subjective perceptions and preferences. Moreover, in households with cohabiting partners, this decision-making process involves certain compromises where financial incentives, interests, gender and power dynamics play an important role. Since educational attainment is positively related to labour market outcomes, such as employment and wages, while at the same time more women are participating in education and the labour market, the gender conflict on the division of work and time within households intensifies and traditional gender roles are challenged. WLB is at the heart of this conflict operating as a mechanism through which division of work and time is reconciled on the individual and household level. Results from the current article reveal great heterogeneity between the 17 European countries examined. Perhaps surprisingly, educational attainment can have a detrimental effect on the WLB of spouses and cohabiting partners, especially for women whose level of WLB seems also more sensitive to fluctuations of the macro-economic climate of the country they live in. However, there is an indication that when an economy goes into recession, higher education has a cushioning effect on female’s WLB compared to relatively better economic times.

## 1 Introduction

Many studies indicate that over the last decades the male-breadwinner model in Europe has been declining, while the dual-earner model gains momentum (Gornick & Meyers, 2009; McGinnity & Whelan, 2009; Ochsner & Szalma, 2017). However, there is evidence that more equal participation of women in the labour market has neither changed people’s perceptions of gender equality significantly nor has it improved much the way unpaid work, such as housework, is divided among couples within households (Grunow & Evertsson, 2016, 2019; Hofacker & König,2013; Ochsner & Szalma, 2017; Steiber, 2009; Wallace, 2017).

The conceptual framework of this research mainly revolves around existing theories pertinent to labour division in households. Additionally, it is also tangential to theories on work–life balance (WLB). Thus, for clarity, the most relevant theories of both streams will be presented; however, the context of this research lies much closer to the labour division theories rather than the one related to WLB, and therefore prime attention is given to the former.

Regarding existing theories on labour division, a stream of literature argues that although inequalities among the classic socio-economic factors of social stratification, such as education and class are persistent, they are manifested in different ways across countries. Contrariwise, standard economic theoretical approaches tend to neglect the role of contexts or imply that contexts across countries do not differ substantially (Brines, 1994; Crompton, 2006; Fagan, Lyonette, Smith, & Saldaña-Tejeda, 2012; Wright, 1997). While literature is abundant on the positive effect of education on employability and wages for both genders, its relationship with WLB is not that straightforward to interpret (Dotti Sani & Scherer, 2018; Kalleberg, 2011; Kromydas, 2015; Steiber, Berghammer, & Haas, 2016).

Becker’s (1981) rational choice approach to the family is considered as a landmark in family economics. Essentially Becker, departing from Mincer’s (1958) human capital theory, sees no real difference in decision making processes between individuals, households, firms or countries where perfect equilibrium is eventually succeeded through utility maximisation where resources are perfectly allocated among individuals or groups such as households. Full information on each member’s comparative advantage, opportunity costs and task specialisation is assumed. Eventually, this leads to optimal outcomes not only on the individual but also on the household level. Consequently, the gendered division of labour is determined by differences in comparative advantages and specialisation and are independent of power relations and women’s exploitation from men. Although Becker acknowledges that such exploitation exists, it is not seen as a barrier for an efficient division of labour within a household since, when women have no apparent comparative over men in childcare and house-work, there is no economic incentive for a division of labour based on gender.

The bargaining theory, on the other hand, acknowledges that within households personal and households’ interests can be conflicting and thus bargaining power prevails over all other factors. There is no diversion from human capital theory basic notions of utility maximisation and rational decision making; however its theoretical base is more informed by individual choices and interests, which can in turn conflict with some household’s goal as an economic entity (Coltrane, 2000; Crompton, 2006). Time-allocation within households is a decision-making process, where individuals use their bargaining power to split a predetermined amount of time into time allocated to either work or leisure (Bianchi, Milkie, Sayer, & Robinson, 2000; Coltrane, 2000; Crompton, 2006; Heisig, 2011; Lundberg & Pollak, 1996; Parsons, 1949).

A different stream of research challenges approaches from economics by shifting the focus on gender roles, perceptions, attitudes and expectations regarding cultural and other societal norms, manifested in the form of gender ideologies that influence individual decision-making processes (Braun, Lewin-Epstein, Stier, & Baumgärtner, 2008; Deutsch, 2007; Heisig, 2011; Pfau-Effinger, 2004). For example, certain time allocation decisions taken in a household context are not always based on equity and fairness (Pahl, 1984; Wallace, 2017). Power relations and social roles that are defined by gender stereotypes can also dictate time allocation and labour division within couples. As a result, the dominant paradigm prevails and, therefore, inequality persists. Inequality especially propagates where task specialisation becomes socially biased, leading to women being in a subordinate position as they are economically dependent on men (Brines, 1994; Lewis, 1992; Sullivan, 2004). Moreover, the ‘doing gender’ approach coined by West and Zimmerman (1987) treats gender as a social construction. Gender differences are not just natural or biological. The gendered division of work is propagated in public discourses and practices where economic rationality is amalgamated by instrumental and moral factors, which in turn can change during the life course (Duncan, 2005; Naldini & Solera, 2018; West & Zimmerman, 1987). Hakim (2000) focuses on preferences instead, arguing that, at least in modern Western societies, women’s choice between working and committing to the household is simply a matter of preference.

Coming to theories on WLB, a number of theoretical models have been developed in the literature. The most common are the ecological systems theory, the positive psychology and the Job Demands-Resources (JD-R) theory. The ecological systems theory essentially treats WLB as a multilevel concept where all levels (micro, meso and macro) are constantly interacting and can be equally facilitative or conflictive (Bronfenbrenner, 1979; Kromydas, 2017). Then, positive psychology focuses more on positively-oriented organisational behaviour, human resource strengths and psychological capacities. This theory is oriented more towards the micro and meso level (Seligman & Csikszentmihalyi, 2014). Finally, the JD-R theory defines WLB as the best fit between resources and demands across work and family domains (Bakker & Demerouti, 2007). Unfortunately, in all these theories the gender dimension and context in the macro level are essentially overlooked. Even though the ecological systems theory implies a relationship between WLB and context in the macro level, it is unclear what the direction of this relationship is and whether people with different characteristics, such as gender, are affected alike.

Proving or disproving a specific theoretical framework is not the main scope of the current article. Given the wealth and breadth of theoretical models on WLB and the gendered division of labour, such an attempt would have been seriously biased and highly selective. Instead, an alternative, more inclusive approach was followed, where the relationship between the WLB of men and women spouses and cohabiting partners and their educational attainment is empirically tested in a two-step regression analysis stratified by gender and time. WLB is represented by a composite binary indicator for perceived WLB that focuses on the work-side interference into private life. This is used as the outcome variable in regression analysis. The main predictor variable is years of educational attainment and its statistical association with the WLB indicator is explored separately in 17 European countries. Given the lack of a gender dimension on WLB conceptual frameworks, the current article places the concept of WLB within the broader domain of the gendered division of labour by employing a quantitative strategy that, apart from human capital, can arguably accommodate a number of structural elements of various theoretical models related to WLB and gendered division of labour. These elements are represented by specific proxies (variables or block of variables) that are used as controls in the form of effect moderators to reveal the effect of education on WLB and also whether this differs by gender and countries’ macro-economic climates (see Table A1 in the Supplementary File).

The next section of this article reviews the relevant literature. Then, the data and methods used are explained followed by an interpretation and illustration of the results. The article concludes by critically discussing the results in relation to the existing literature and their implications for policymaking.

## 2 Literature Review

Moving beyond the individual level, existing literature argues that within households a higher-educated male who cohabits with a heterosexual partner is more likely to be involved in more housework compared with a lower-educated one. Hence, given that couples are usually educationally matched, especially in economically developed countries, women within couples that are higher educated, spend less time on housework compared to the lower-educated ones (Coltrane, 2000; Gershuny, 2000; Oinas, 2018). Nevertheless, as with paid work, a specific gender pattern seems to exist that categorises types of housework as ‘masculine’ or ‘feminine’ even in high-income gender-egalitarian regimes, such as the Nordic countries (Tammelin, 2018b). While there is some indication of a gender convergence in the amount of time male and female cohabiting partners spend on housework, gender segregation in domestic tasks as reinforced by specific gender ideologies and stereotypes, remains a significant obstacle for achieving an equal division of labour (in terms of both paid and unpaid work) in heterosexual couples (Kan, Sullivan, & Gershuny, 2011).

Previous research has indicated that long hours of paid work for men reinforces the male-breadwinner paradigm. However, this is true when their female partners are working long hours as well (Ciccia & Bleijenbergh, 2014). In that case, the physical and psychological burden for women to balance long hours of paid work and unpaid housework is enormous. Certainly, well-structured public childcare and parental leave systems decreases the burden of housework on women, incentivising them to become more active in the labour market. At the same time, the greatest proportion of parental leaves are taken by women, indicating a social prejudice against them as, in practice, childcare is widely considered as a rather ‘feminine’ task (Tammelin, 2018b). Thus, generous public childcare policies themselves are important but not enough to tackle gender inequality within households as they need to be accompanied by a culture shift towards more egalitarian perceptions on gender where men share the housework/childcare burden more equally with their female partners.

The literature on the indicators used to capture WLB, regarding geographical and cultural differences, is very limited. The indicators currently used do not include mechanisms with which differences between countries of different levels of economic development or welfare structures can be captured. Furthermore, the focus is rarely on educational or gender differences, albeit considerable evidence showing that women, especially the lower-educated, hold job positions with high levels of insecurity while working unsocial hours and in precarious industries such as call centres and hospitality (Gautie & Schmitt, 2010; Ghai, 2003; Stier &Yaish, 2014). Individual WLB preferences are more straightforward to be defined, but research on the household level and the effect of institutional factors is limited (Bianchi & Milkie, 2010; Korpi, Ferrarini, & Englund, 2013). Our article attempts to fill these gaps focusing on the relationship between educational attainment and gender division of labour within families, as empirically instrumented by a composite binary indicator for perceived WLB that focuses on the work-side interference into private life. Moreover, the 17 European countries examined are classified under the welfare state regime they belong to, according Esping-Andersen (1990), Ferrera (1996), Fenger (2007), Arts and Gelissen (2010) and Gallie (2013). However, this is only for illustrative purposes, to identify whether there are similarities or differences between countries. The current research acknowledges that the traditional welfare state regime classification is regarded outdated by the most recent literature, as it does not entirely reflect the current reality of family policies and the gender division of labour. More recent developments on the welfare regime literature include a gender perspective, while others challenge the traditional welfare regime classification (especially the Southern and Eastern regimes) in relation to the gender roles they represent since family policies, but also the economic activity rate of women and the incidence of full-time work and dual-earner couples differ significantly across countries (Esping-Andersen, 2009; Saxonberg, 2013; Wall & Escobedo, 2013).

Table 1 replicates a table found in Tammelin (2018b, p. 14). It illustrates three conceptual models on the division of work in families. The male breadwinner model implicitly or explicitly accepts separate roles for each gender where only men are active in the labour market and women do unpaid work (Parsons, 1949; Treas & Drobnič, 2010). Therefore, in societies where such perceptions exist, policies that aim to increase labour market participation for women might have adverse consequences. Instead of alleviating work–life conflicts, they might channel women into jobs that are part-time, temporary and, therefore, low-paid with low-levels of security, leaving the good jobs and career laddering to men, while strengthening and reproducing the traditional role of men as breadwinners and women as mainly being responsible for childcare and household chores (Tammelin, 2018a). The ‘moderated’ version of the traditional model is the caregiver parity model, where traditional gender roles persist within the household; however, women and men are treated more equally in terms of labour market participation. Although this model (either in its core or moderated version) seems more common to Continental, Southern and Eastern Europe, there are specific countries within these country groups that significantly differ within each other in the implementation of parental leave and childcare policies (Saxonberg, 2013; Wall & Escobedo, 2013). The second stream reflects a framework with a relatively low degree of policy interventions where family issues, such as housework or child-care, are outsourced either to professionals or to relatives. In this case, issues such as the WLB within couples are reconciled more by common agreements between partners domestically, and less by policymaking and related incentives. This is the universal breadwinner model and is mostly associated with the Anglo–Saxon countries; however, recent evidence shows that it can be found in countries such as the Netherlands or Portugal (Wall & Escobedo, 2013). Finally, the third model, known as the Nordic model, concerns an egalitarian culture for paid work and housework as well as caring responsibilities. Some authors argue that in terms of childcare and parental leave policies, Norway and Finland might divert from this model, resembling more to countries in central Europe such as France and Belgium, while others claim that this model does not find application to any country and still remains a utopia (Tammelin, 2018a; Wall & Escobedo, 2013).

Still, welfare regime classifications are very sensitive to the data and the criteria used. Even if more egalitarian childcare and parental leave policies are aiming towards a more gender equal division of work, they are by no means sufficient if not accompanied by similar individual attitudes, behaviours and perceptions. Undoubtedly, policies and perceptions relate to each other but causality in this relationship is still unclear. In any case, this is beyond the scope of the current article, as country classification in welfare regimes has not been used for explanatory but rather for illustration purposes.

Yates and Leach (2006) argue that reforms promoting work flexibility have increased negativity among workers, as well as anger and introversion. Moreover, there has been a continuous decline in workers’ willingness to look after their families and to actively participate in communities and this, eventually, has led to an increase in social exclusion. Such a situation is likely to worsen during a recession. Part-time work, temporary employment agency assignments, flexible employment, short-term and contingent work and independent contracting are all examples of non-standard employment that can increase uncertainty and the feeling of job insecurity (Kalleberg, 2011). These are the main employment arrangements that have become increasingly debated in recent years, gradually shaping current trends in modern employment in relation to cultural, institutional and regulatory societal norms. These arrangements demarcate a reorientation in the conceptualisation of work and employment and, along with this, that of WLB (Eurofound, 2017).

Women are disadvantaged in the labour market, having on average lower wages compared to their male counterparts. Women also work, on average, fewer paid hours and usually do more housework than men (Gautie & Schmitt, 2010). However, it remains unclear whether this leads to lower or higher levels of reported WLB compared to men, especially within households. According to past research on this topic, this also depends on factors such as the number of children living in the household, income levels, employment status, occupation and industry, the amount of working hours, aspects of job quality on regularity and intensity of working life or the identification of clear boundaries between working life and non-working life, and also public attitudes and perceptions regarding gender equality (Anttila, Oinas, Tammelin, & Nätti, 2015; Crompton & Lyonett, 2006; Fagan et al., 2012; Gallie & Russell, 2009; Hofacker & König, 2013; McGinnity, 2014; Muñoz de Bustillo, Fernandez-Macias, Anton, & Esteve, 2009; Russell & McGinnity, 2013; Tausing & Fenwick, 2001; Wallace, 2017). This article employs a methodological strategy that accounts for these factors by using them in regression models as predictors in the form of control variables. Even if educational attainment is a very important factor that is positively related to labour market outcomes its relationship with WLB is essentially neglected in the literature. Human capital theory and its application to the household level by Becker (1981) treats education as an investment that finds application only to paid work. It is possible though that educational attainment affects practices on the individual as well as on the household level with respect to unpaid work as well, triggering WLB gender differences within the same household. The economic climate is also likely to moderate such effects differently as gender roles might become of a lower importance when the economy dives into a deep recession like the one in 2008, or perhaps the one that is currently looming due to the COVID-19 outbreak. In most European countries, the economic crisis of 2008 triggered a vicious economic downward spiral. People with lower educational qualifications have been affected the most, both in terms of employment and pay (Gallie, 2013; Hurley, Enrique, & Storrie, 2013). However, little attention has been paid to how this has affected WLB on the individual but also on the household level, where gender differences might appear. The current article aims to contribute to the relevant literature by investigating whether the effect of educational attainment on the WLB of ESS respondents who cohabit with a heterosexual partner differs by gender and also across countries with different macro-economic climate, controlling for various variables identified in the literature as determinants of WLB (see Table A1 in the Supplementary File).

## 3 Data and Methods

The current research uses individual-level data from the European Social Survey (ESS), rounds 2004 and 2010, focusing on 17 European countries. The countries included in the analysis are Belgium, the Czech Republic, Germany, Denmark, Estonia, Spain, Finland, France, the UK, Greece, Ireland, Netherlands, Norway, Poland, Portugal, Slovenia and Slovakia. The ESS is a biannual survey that aims to capture socio-economic attitudes and values in Europe. Survey questions regarding WLB, working conditions and pay can provide useful insights into respondents’ perceptions of their WLB before (ESS Round 2, 2004) and during (ESS Round 5, 2010) the most recent 2008 economic recession. Men and women in paid employment, aged 25 to 70 years old, living with their partners at the time they were interviewed are included in the analysis. Self-employed were excluded due to the very different nature of work and related WLB patterns. Moreover, educational attainment is measured in years of education representing the years of attained education below or above the compulsory level in each country examined. Based on past literature relevant to WLB determinants (Anttila et al., 2015; Crompton & Lyonette, 2006; Fagan et al., 2012; Gallie & Russell, 2009; Hofacker & König, 2013; McGinnity, 2014; Russell & McGinnity, 2013; Tausing & Fenwick, 2001; Wallace, 2017), a binary index representing perceived WLB focusing on the work-side interference into private life has been constructed combining the following five variables: (1) *work involves working evenings/nights*; (2) *work involves having to work overtime at short notice*; (3) *work involving working on weekends*; (4) *job prevents you from giving time to partner/family*; (5) *how often do you feel too tired after work to enjoy things you like to do at home?*

Before this index was constructed, all five components were also dichotomised, where the value of 0 corresponds to low levels and 1 to high levels of WLB, meaning that those whose responses include three or more 1 were classified as having a job with high levels of WLB and vice versa. Apart from educational attainment, the final models estimated include other factors that are empirically known in the literature as determinants of WLB. The statistical effect of all these factors is presented in the Supplementary File (Tables A3–A6).

The analysis was performed in two steps. The first step concerned multivariate regression analysis and, particularly, logistic regression models in a fixed-effects format. Effects are presented in the form of Odds-Ratios (OR). Robust standards errors were used to account for heteroskedasticity and clustering of observations. Design and population weights were used as recommended by ESS (Kaminska, 2020). In the first step, three models were estimated and stratified by gender (six in total). The assumption made is that WLB is a function of *X*_*i*_ variables (including the interaction term), commonly used in the literature as potential factors that can affect WLB on the individual level. In Table 2, Models 1a for males and 1b for females refer to the pooled dataset (2004 and 2010). The two models include all control variables and an interaction term between the variables that represent country (*C*_*i*_) and calendar year (*T*_*i*_), estimating how WLB levels have changed from 2004 to 2010 (hereafter called ΔWLB).

Particularly, for an individual *i*, Models 1a and 1b are represented by Equation 1:

(1) $$\begin{array}{cc} \begin{array}{l} {\text{WLB}}_{i} \end{array} & \begin{array}{l} =a+\text{exp}\left(b_{1}\right)YEd_{i}+\text{exp}\left(b_{2,4,5\ldots 19}X_{i}\right)+\\ +\text{exp}\left(b_{20}\right)C_{i}+\text{exp}\left(b_{21}T_{i}\right)+\\ +\text{exp}\left(b_{22}T_{i}C_{i}\right)+\varepsilon_{i} \end{array} \end{array}$$

Here, *X* is a vector of 19 control variables, *T*_*i*_ *C*_*i*_ denotes an interaction term between calendar year and country and *YEd*_*i*_ years of educational attainment centred at the compulsory level in each country. In the same equation interaction’s constitutive terms are also included, representing the effect of the one term when the other is on its reference category. In terms of the country variable, the Netherlands has been selected as the reference category because it is the country with the highest levels of WLB on average for both genders in 2004 and 2010 and therefore *C*_*i*_ shows OR differences from the highest performing country in terms of WLB in 2004, while the interaction shows OR differences again from the Netherlands in 2010. For the *T*_*i*_ variable the reference category is 2004. The OR for *T*_*i*_ shows how much higher or lower the odds of having a job with high levels of WLB (WLB = 1) are in the Netherlands in 2010 compared to 2004. Thus, it shows ΔWLB for the Netherlands only. The effect of the interaction term shows how much the effect of living in 2010 on WLB differs between the Netherlands and other countries. Then, since this is a logistic regression where OR are calculated and relationships between variables take a multiplicative form, the product of the country variable and the interaction term (*C*_*i*_ × *T*_*i*_ *C*_*i*_) shows how much the odds of having a job with high levels of WLB in 2010 change compared to 2004 for each country separately, which is the ΔWLB term, mentioned above. For Models 2a, 2b and 3a, 3b, represented by Equations 2 (2004) and 3 (2010), apart from all control variables, an interaction is also included between *YEd_*i*_* and *C*_*i*_ (*YEd*_*i*_ *C*_*i*_) using the same reference categories as in (1) to compare the effect of education across the 17 countries examined. Similarly to Equation 1, the constitutive terms of the interaction are also included. Since regressions are run for 2004 and 2010 separately, *T*_*i*_ is now missing from Equations 2 and 3. The interaction shows the effect of an additional year of education on WLB in the reference category separately for men (2a, 3a) and women (2b, 3b). The effect for each country is then calculated through a multiplication between the interaction term and each value of *C*_*i*_ that represents countries (*C*_*i*_×*YEd*_*i*_ *C*_*i*_). MWLB_ed_ refers to the value of *C*_*i*_×*YEd*_*i*_ *C*_*i*_ for males while the equivalent notation for female is FWLB_ed_. The *ε*_I_ represents the error term in all three equations.

(2) $$\begin{array}{cc} \begin{array}{l} {\text{WLB}}_{2004i} \end{array} & \begin{array}{l} =a+\text{exp}\left(b_{1}YEd_{i}\right)+\text{exp}\left(b_{2,4,5\ldots 19}X_{i}\right)+\\ +\text{exp}\left(b_{20}C_{i}\right)+\text{exp}\left(b_{21}YEd_{i}C_{i}\right)+\varepsilon_{i} \end{array} \end{array}$$

(3) $$\begin{array}{cc} \begin{array}{l} {\text{WLB}}_{{}_{2010i}} \end{array} & \begin{array}{l} =a+\text{exp}\left(b_{1}YEd_{i}\right)+\text{exp}\left(b_{2,4,5\ldots 19}X_{i}\right)+\\ +\text{exp}\left(b_{20}C_{i}\right)+\text{exp}\left(b_{21}YEd_{i}C_{i}\right)+\varepsilon_{i} \end{array} \end{array}$$

In the second step, all OR that correspond to the two aforementioned statistical interactions are regressed in a bivariate manner over three variables that can arguably represent a country’s economic climate. These are the GDP growth and unemployment rate, which are the indicators most commonly used in the literature, to define whether an economy is an expansionary or recessionary business cycle (National Bureau of Economic Research, 2010). The relationship between estimations from the first step and the three macro-economic indicators used is presented illustratively in graphs. Graphs are drawn only for the relationships that are statistically significant. Their actual effect size and associated statistical significance are presented in the Supplementary File (Table A5). Because of the dynamic nature of these two macro-economic indicators, it was decided that single-year comparisons (i.e., 2004 vs. 2010) are unsuitable to capture this effect, and therefore, four-year averages prior to 2004 and 2010 were used (Ostry, Berg, & Tsangarides, 2014). Additionally, a variable that shows the subjective judgements of ESS respondents on the state of the economy in their residence country is also used for 2004 and 2010. This variable has values from 0 to 10 where 0 reflects complete dissatisfaction and 10 complete satisfaction. In this way, perceptions of the economic climate were also captured. This variable has been aggregated on the country level for the purposes of the analysis.

## 4 Results

Table 2 shows all three interaction’s effects as explained in Section 3. Results from Model 1a indicate that reported WLB for both genders do not follow a consistent pattern across the welfare state regimes. WLB in the Nordic countries increases from 2004 to 2010 for both genders, apart from females in Norway, where their WLB is marginally lower compared to 2004. Then, in the Anglo-Saxon countries, WLB increases in the UK for both genders in a rather balanced manner, whereas in Ireland it decreases slightly for men and considerably for women. In the rest of the countries WLB falls for both genders in 2010 compared to 2004, with the exceptions of Portugal, Slovenia, Estonia and Slovakia where it increases only for males and Poland, Slovakia, the Netherlands and Spain only for females. With regards to the variable that shows years of educational attainment, when differences between countries are not taken into account, it was statistically insignificant for males while, for females, it was significant but negatively correlated, implying that higher educational attainment is a disinvestment to their WLB levels (the term is not shown in Table 2, as it is part of the vector of control variables [*X*_*i*_] in Equation 1, but its estimation can be found in the Supplementary File, Table A3).

Models 2a and 3a in Table 2 account for cross-country differences in educational attainment between 2004 and 2010 through an interaction between years of education and country, which was jointly significant for both genders and years. However, the effect is rather small in most countries implying that educational attainment is not such a strong determinant of WLB. Looking at males in 2010 compared to 2004, the effect remained or became positive in the Netherlands from the Continental countries, in none from the Southern countries, in Estonia and Slovakia from the Eastern countries and in Norway from the Nordic countries. For females, the effect remained or became positive only in Ireland, Norway, Finland and the Czech Republic, suggesting that in most countries WLB was negatively affected by educational attainment. In general, WLB is even more weakly identified by educational attainment in 2010 compared to 2004 for both males and females, especially for females.

**Figure 1:**
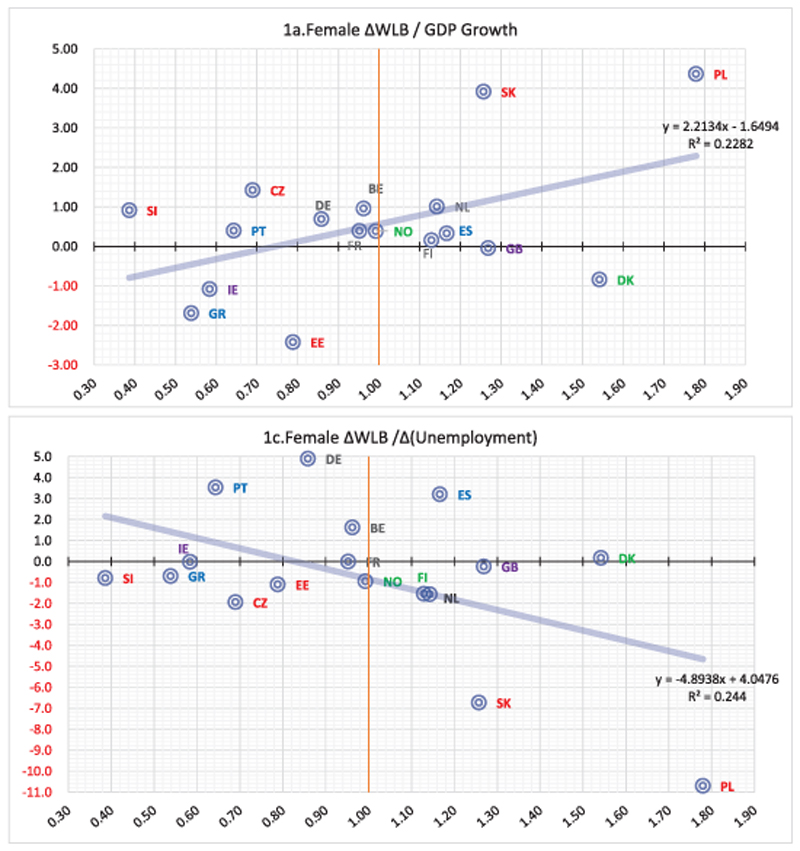

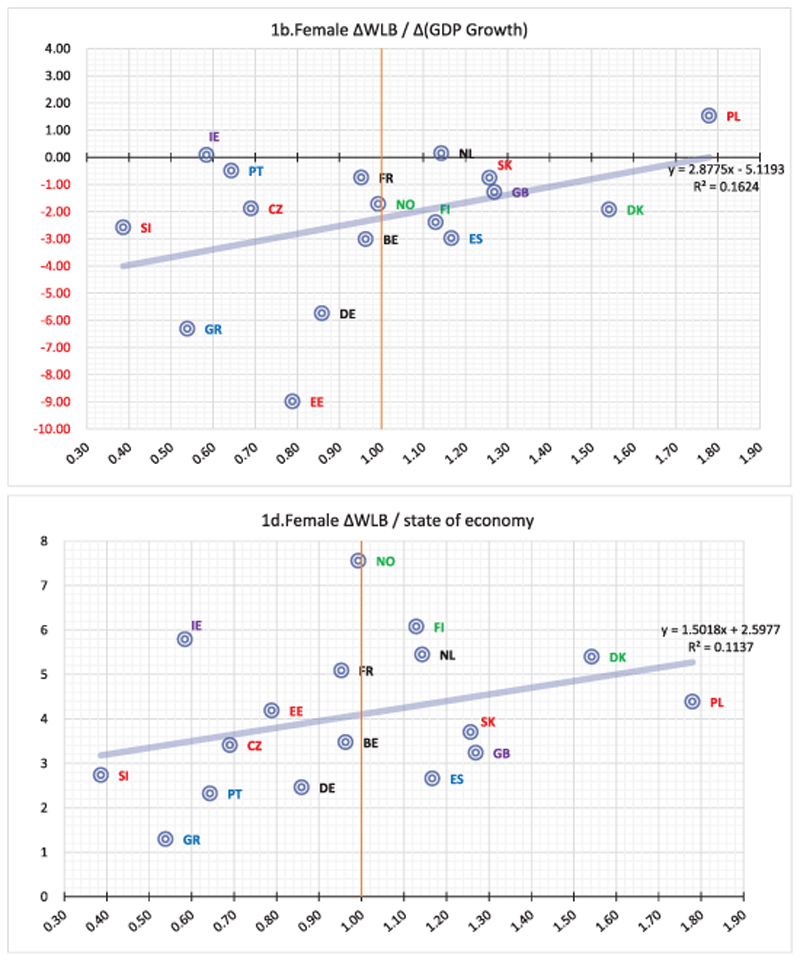
Statistically significant bivariate linear regressions (Female ΔWLB, second step). ΔWLB denotes difference in WLB between 2004 and 2010, Δ denotes difference. Statistically significant bivariate linear regressions (Female ΔWLB, second step). ΔWLB denotes difference in WLB between 2004 and 2010, Δ denotes difference.

Results for the second step of the analysis showed a conflict between genders, since, when the GDP growth in a country is relatively high, WLB for female tends to follow suit as Figure 1a shows. For men, the equivalent statistical effect is insignificant. In terms of temporal changes from 2004 to 2010 (Figure 1b) for women, an increase in GDP could have a positive effect on their WLB. The unemployment rate itself seems unrelated to WLB; however, women seem more sensitive to unemployment temporal changes, since a temporal decrease (increase) in the unemployment could lead to an improvement (deterioration) of their WLB (Figure 1c). Similarly, females that live in countries with, on average, more positive perceptions on the state of the economy enjoy higher WLB levels (Figure 1d). For men, all the above relationships were estimated as statistically insignificant, implying that economic climate is not associated with how their WLB levels are determined.

**Figure 2:**
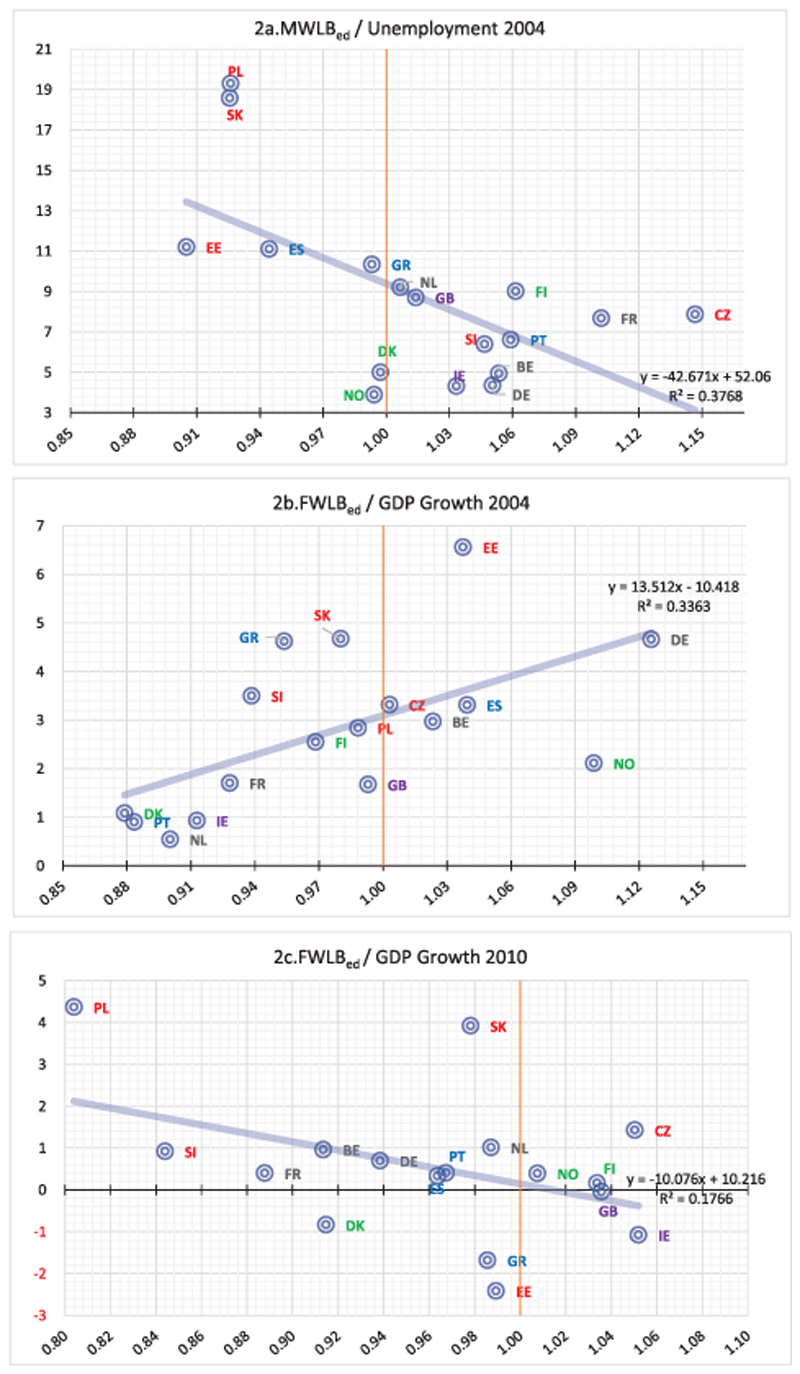
Statistically significant bivariate linear regressions (MWLB_ed_ and FWLB_ed_ 2004 and 2010, second step). MWLB_ed_ denotes the effect of an additional year of educational attainment for males who cohabit with female partners. FWLB_ed_ is the equivalent notation for females who cohabit with a male partner.

The effect of educational attainment on WLB seems diverse among countries and between genders. In 2004, high educated males compared to lower-educated, are better (worse) off in terms of WLB in countries with low (high) unemployment (Figure 2a). For females who cohabit with male partners, unemployment rates do not affect FWLB_ed_ but its relationship with the GDP growth rate is statistically significant in both years examined (Figures 2b and 2c). Yet, it appears that whereas in 2004 (Figure 2b) in countries with relatively higher GDP growth rates, FWLB_ed_ was also higher, the relationship becomes negative in 2010 (Figure 2c). This bi-directional relationship across time implies that the associations among educational attainment, WLB and GDP is not that straightforward to interpret and might be attributed to other unob-servable confounding factors. Perceptions of the state of the economy were found insignificant for both genders and years.

**Figure 3:**
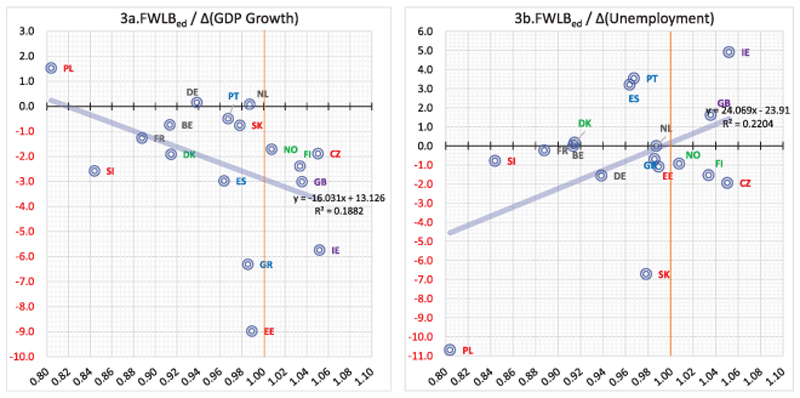
Statistically significant bivariate linear regressions (MWLB_ed_ and FWLB_ed_ 2004 and 2010 Temporal changes, second step). FWLB_ed_ denotes the effect of an additional year of educational attainment for females who cohabit with a male partner.

Regarding the GDP growth and unemployment rates’ temporal changes between 2004 and 2010, an additional year of education leads to an improvement of WLB for females when GDP growth rates fall (Figure 3a), and unemployment increase (Figure 3b). Thus, when a country moves from growth to recessionary periods, education has a rather ‘cushioning’ effect on females’ WLB. However, in most countries FWLB_ed_ < 1 and therefore educational attainment is still a drawback rather than an advantage for their WLB. The effect in recessions is still negative, but rather weaker compared to high-growth, low-unemployment economic times. Comparing these results with those in Models 1b and 2b and considering that dual earner couples are gradually becoming the norm as well as that the 2008 recession halted full-time employment growth exacerbating the creation of part-time and atypical jobs that are more likely to be taken by low-skilled women, then the above ‘diminishing’ negative effect seems plausible, but also calls for further research to generate knowledge that can be used in future recessions in the form of mitigating measures. Moreover, the relationship between the 2004–2010 temporal changes in subjective judgments on the state of economy and MWLB_ed_ or FWLB_ed_ levels is statistically insignificant.

Finally, for sensitivity analysis purposes six gender models have been constructed, as in Steiber et al. (2016), based on specific ESS variables that refer to both respondents’ and partners’ amount of working hours, employment status and employment mode (part-time or full-time). Using the total average across all 17 countries as a threshold, it appears that there is no clear welfare regime pattern that holds in both 2004 and 2010 for all countries apart from the Nordic group (perhaps with the exception of Norway) where the dual-breadwinner is dominant, and the male-breadwinner model is weak (see Table A2 in the Supplementary File). Yet, in all 17 countries, women spend consistently much more time than men in housework activities. In line with Tammelin (2018b), the supplementary analysis performed showed that the difference is large and statistically significant in all countries indicating that in reality there is no such a thing as a Universal caregiver model. In Nordic countries though the difference in the mean hours spent on housework is smaller but still significantly different between men and women.

## 5 Conclusion

Results suggest that the effect of education on WLB is diverse across the 17 European countries examined, but in most cases, it is weak for both genders. In most countries the effect in 2010 turns negative, especially for women. In terms of welfare state regimes, no common temporal pattern has been identified. These results are in line both with Gallie and Russell (2009) and Strandh and Nordenmark (2006), who argue that production regimes or welfare institutions of a country cannot explain how WLB and the division of labour between paid and unpaid jobs can be determined within households,and with Ciccia and Bleijenbergh (2014) and Tammelin (2018a), who claim that gender models are not distinct across countries and welfares state regimes; they rather coexist, even within the same country. Moreover, a considerable heterogeneity is observed on the country level, as no consistent temporal pattern was observed on how educational attainment affects the WLB of males and females. This heterogeneity seems to persist even when results are displayed over more recent welfare state classifications such as those found in Saxonberg (2013) and Wall and Escobedo (2013) where the type of childcare and parental policies are taken into account.

Research is still limited on the determinants of the division of labour and WLB gender differences within households when the economic climate deteriorates. The current research addressed this gap by including three country-level measures. Looking on individuals who cohabit with a heterosexual partner, GDP growth rates are positively related to the WLB of females but not for males, while the former were also more likely to improve their WLB when unemployment was falling. When educational attainment was taken into account, there was no specific pattern for both genders that was significant in both 2004 and 2010; however, for 2004 FWLB_ed_ was likely to be stronger in countries with relatively higher GDP growth, but for 2010 the direction of this relationship changes. When temporal changes between 2004 and 2010 are examined, there is an indication that when an economy goes into recession, higher education has a cushioning effect on female’s WLB compared to relatively better economic times.

Moreover, the analysis performed by this article showed that Becker’s application of human capital in households is rather problematic. The effect of education on WLB is not uniform across gender, countries and different macro-economic climates. Since higher levels of education lead to lower levels of WLB, especially for females, then the human capital theory seems invalid in household arrangements and perhaps theories where gender roles are influenced by perceptions, attitudes and expectations regarding cultural and other societal norms, manifested in the form of gender ideologies that influence individual decision-making processes, are more applicable (Braun et al., 2008; Deutsch, 2007; Heisig, 2011; Pfau-Effinger, 2004).

In strict business terms, numerous studies indicate that a job of good quality and WLB increases productivity (Fields, 2003; Gunderson, 2002; ILO, 2003; Kalleberg, 2011). However, a gender perspective in which women are treated equally to men with respect to not only paid work but also to unpaid work, such as housework, remains absent. Equality should not be restricted within workplaces but should find application within households, as well. Otherwise, gender equality in workplaces could result in widening gender inequalities as a whole.

With regards to policy, European policymakers are not indifferent to identifying the qualitative elements of employment. Although during periods of economic crisis policymaking is directed more towards finding ways to decrease the number of unemployed people, job quality and WLB are also important, as it has close ties with job stability and labour market sustainability (Muñoz de Bustillo et al., 2009). Having a good quality job associated with good WLB can significantly boost people’s sense of well-being. Moreover, well-being is closely associated with sustainability, equality, economic development and standard of living and therefore good levels of WLB can improve these indicators, as well.

In European policymaking agendas, WLB and gender equality are placed very high. However, in essence, little progress has been made on improving job quality and the WLB, particularly for women. Instead, female participation in the labour market seems to increase, while at the same time WLB arrangements in the household level become more complicated, as even if traditional gender roles are constantly becoming obsolete in the labour market, it is still unclear if this stands with the division of unpaid work within households. At the same time, childcare provision and long parental leaves are indeed helpful for couples; however, if not implemented wisely, they could implicitly incentivise and perpetuate the male-breadwinner model. Certainly, such policies promote equality, but they could become more effective if they were also aiming at cultivating a public understanding that the male-breadwinner model is no longer sustainable. Moreover, technological evolutions in the labour market make gender division in job tasks rather indistinguishable. Unfortunately, attitudes and perceptions within households on labour division have not evolved at the same pace. This creates a significant barrier for women, who cannot exploit their full potential even though relevant technological means, certainly exist.

This research was conducted during a period where homeworking arrangements were quite limited across all European countries examined. However, the outbreak of COVID-19 pandemic at the beginning of 2020 and the associated social distancing and lockdown measures are likely to make working and home environments less distinct and thus WLB might need to be examined under a different conceptual framework where home-working is considered a mainstream practice. Certainly, social distancing is rapidly transforming working arrangements and household relationships on many levels. The division of work among household members enters a new era of conflict, where boundaries are extremely hazy, and this poses huge challenges for future research related to WLB, where new theoretical developments are expected to emerge.

In conclusion, the division of labour among couples of different genders and decisions on WLB seems to be determined by arrangements made on the household and not on the country level or even the gender model each country can be classified under in the relevant literature. Moreover, women appear to be more sensitive than men are to negative changes in the economic climate. In most countries, educational attainment is not beneficial in terms of WLB.

## Supplementary Material

Supplementary File

## Figures and Tables

**Table 1:** The division of work in families: Ideal types.

Division of work	Traditional model		Universal breadwinner model (or adult-worker model*	Universal caregiver model**
	Male breadwinner model	Caregiver parity model		
Gender roles	Separate gender roles	Traditional gender roles persist but are more equal	Men’s and women’s equal engagement in the labour market	Equal roles; transforming gender roles inside and outside labour markets
Labour market Outcomes	Males are in paid work Women are not in paid work	Males are in paid work Women are not in paid work (temporarily or long term) or they work part-time hours	Both men and women are in paid work; women are the main carers (dual or triple burden)	Both men and women are in paid work; both do care work Families with long part-time hours

Notes: *Lewis and Giullari (2005); **Crompton (1999), Gornick and Meyers (2009). Table based on Fraser (1994) and Tammelin (2018b, p. 14).

**Table 2:** Odds-Ratios estimations for the interaction between country and years of educational attainment variables in Models 1, 2 and 3.

	MODEL 1a—ΔWLB				MODEL 2a—WLB_ed_—2004				MODEL 3a—WLB_ed_—2010			
Countries	MALE		FEMALE		MALE		FEMALE		MALE		FEMALE	
	ΔWLB (***)	Robust S.E	ΔWLB (***)	Robust S.E	MWLB_ed_ (***)	Robust S.E	FWLB_ed_ (***)	Robust S.E	MWLB_ed_ (***)	Robust S.E	FWLB_ed_ (***)	Robust S.E
Continental												
Belgium (BE)	0.77	[0.041]	0.95	[0.041]	1.11	[0.013]	0.95	[0.027]	0.98	[0.014]	0.92	[0.021]
Germany (DE)	0.78	[0.033]	0.83	[0.066]	1.02	[0.013]	0.92	[0.028]	1.00	[0.021]	0.94	[0.006]
France (FR)	0.89	[0.064]	0.88	[0.060]	1.01	[0.011]	0.99	[0.026]	0.96	[0.012]	0.89	[0.015]
The Netherlands (NL)	0.67	[0.016]	1.21	[0.093]	1.05	[0.014]	0.91	[0.018]	1.00	[0.105]	0.99	[0.013]
Southern												
Spain (ES)	0.99	[0.055]	1.23	[0.062]	0.95	[0.015]	1.04	[0.032]	0.98	[0.009]	0.96	[0.008]
Greece (GR)	0.76	[0.107]	0.48	[0.031]	0.99	[0.021]	0.92	[0.028]	0.97	[0.015]	0.99	[0.014]
Portugal (PT)	1.53	[0.051]	0.64	[0.049]	1.07	[0.015]	0.88	[0.029]	0.90	[0.009]	0.96	[0.022]
Eastern												
The Czech Republic (CZ)	0.55	[0.044]	0.64	[0.044]	1.15	[0.026]	1.00	[0.036]	1.00	[0.043]	1.05	[0.018]
Estonia (EE)	1.59	[0.070]	0.85	[0.081]	0.91	[0.023]	1.05	[0.051]	1.08	[0.029]	0.99	[0.016]
Poland (PL)	0.74	[0.042]	1.71	[0.083]	0.92	[0.013]	0.98	[0.055]	0.97	[0.026]	0.81	[0.011]
Slovenia (SI)	2.08	[0.353]	0.38	[0.031]	1.04	[0.021]	0.92	[0.048]	0.99	[0.029]	0.85	[0.022]
Slovakia (SK)	1.05	[0.111]	1.25	[0.075]	0.93	[0.022]	1.01	[0.015]	1.21	[0.045]	0.98	[0.024]
Anglo-Saxon												
Great Britain (GB)	1.31	[0.088]	1.28	[0.086]	1.06	[0.018]	0.99	[0.021]	1.06	[0.013]	1.03	[0.007]
Ireland (IE)	0.98	[0.064]	0.54	[0.046]	1.05	[0.019]	1.12	[0.020]	0.97	[0.013]	1.05	[0.008]
Nordic												
Denmark (DK)	1.44	[0.029]	1.48	[0.098]	1.00	[0.012]	0.87	[0.017]	0.98	[0.008]	0.92	[0.013]
Finland (FI)	1.09	[0.072]	1.17	[0.074]	1.06	[0.013]	0.97	[0.015]	0.94	[0.016]	1.03	[0.013]
Norway (NO)	1.36	[0.086]	0.98	[0.073]	0.98	[0.012]	1.08	[0.009]	1.07	[0.011]	0.97	[0.014]
N	8,374		7,877		3,805		3,496		3,771		3,702	
Pseudo-R^2^	0.18		0.21		0.21		0.24		0.19		0.22	

Notes: ΔWLB denotes difference in WLB between 2004 and 2010, MWLB_ed_ denotes the effect of an additional year of educational attainment for males who cohabit with female partners. FWLBed is the equivalent notation for females who cohabit with a male partner. ΔWLB, MWLB_ed_ and FWLB_ed_ have been found statistically significant at the 99% confidence level. * p < 0.10,** p < 0.05, ***p < 0.01. Asterisks in brackets indicate statistical significance for the interaction (joint F-test). Source: ESS Round 2 (2004) and ESS Round 5 (2010).
