# Supplementary material for: Educational Attainment and Gender Differences in Work–Life Balance for Couples across Europe: A Contextual Perspective
Source: Soc Incl. Author manuscript; Available in PMC 2020 Nov 3. (PMC7116291; doi:10.17645/si.v8i4.2920)
Supplement: Supplementary File [file EMS97687-supplement-Supplementary_File.pdf]

Appendix

Table A1: Control variables used in Models 2 and 3

	Control variables	Theoretical framework	Relevant Literature
1	**Job discretion, autonomy and flexibility** **Values:** (0-4 scale: lowest to highest)	Resource bargaining, JD-R	Bianchi *et al.* 2000; Coltrane 2000; Crompton 2006; Lundberg and Pollak 1996; Heisig 2011, Parsons, 1949; Bakker & Demerouti, 2007
2	**Security of Employment** **Values:** (0: Low security, 1: High security)		
3	**Workplace relationships and work motivation** **Values:** (0-4 scale: lowest to highest)		
4	**Safety in employment** **Values:** (0: Low safety, 1: High safety)		
5	**Job Intensification** **Values:** (0-3 scale: highest to lowest)		
6	**Trade Union Membership** **Values:** (1: No member, 2: Yes currently, 3: Yes previously)	Resource bargaining	
7	**Partner has a higher degree** **Values:** (0:No, 1: Yes)	Resource bargaining/ Homogamy dimension	
8	**Having dependent children living in the household** **Values:** (0: Yes, 1: No)	Time constraints	
9	**Hours worked overtime included** **Values:** (Continuous variable / Mean 2004 40.24:, Mean 2010: 40.24)		
10	**Total time people in home spend on housework, hours typical weekday** **Values: (**Continuous variable)	Time constraints/Household dimension	
11	**Respondent's partner is working** **Values:** (0: Working, 1: Other than working)		
12	**Total hours per week your partner spends in housework** **Values: (**Continuous variable -only in 2010)	Time constraints Partner's situation /Household dimension	
13	**Part your partner spend of total time housework on typical weekday** **Values:** None or almost none, Up to 1/4 of the time, More than 1/4, up to 1/2 of time, More than 1/2, up to 3/4 of time, More than 3/4, less than all of time, All or nearly all of time) -Only in 2004		
14	**Age** **Values:** (Continuous variable / Mean 2004:42.63 , Mean 2010: 43.71)	Not related to any specific framework. However it consists an element to all	
15	**Industry** **Values: (**Agriculture/Fishing, Mining, Manufacturing, Energy, Water Supply Waste, Construction ,Retail, Transportation and Storage, Accommodation and Food, Infor/Communication, Finance, Real Estate, Prof/Sci/Tech Activities, Admin/Support/Serv & Public/Defence/Social Sec, Education, Health and Social Work, Arts, Entertainment and Recreational activities, Other Services)	Social class theories	Brines 1994; Crompton,2006; Fagan 2001; Wright 1997
16	**Occupational category (Wright, 1997)** **Values: (** Expert managers: Expert supervisors, Experts, Skilled managers, Skilled supervisors, Skilled workers, Low skilled managers, Low skilled supervisors, Low skilled workers)	Social class theories	
17	**Hampered by health problem** **Values:** (1: No, 2: Yes to some extent, 3: Yes a lot)	Well-being	Seligman & Csikszentmihalyi, 2014; Tammelin 2018; Oinas, 2018 Kalleberg, 2011
18	**Satisfied with life in general** **Values:** (0-10 scale: lowest to highest)		
19	**Women should be prepared to cut down on paid work for sake of family** **Values:** (Agree, Neither agree nor disagree, Disagree, Disagree strongly)	Perceptions/ Culture/ "Doing gender"	West & Zimmerman 1987; Lewis 1992; Braun *et al.* 2008; Deutsch 2007; Heisig 2011, Pfau-Effinger, 2004

Table A2: Calculated Gender Models prevalence based on ESS data for 2004 and 2010

Countries	Dual-breadwinner[1]		Male-breadwinner[2]		Modernized male-breadwinner[3]		Dual part-time[4] *		Female-breadwinner[5]		No-full-earner[6]	
	**2004**	**2010**	**2004**	**2010**	**2004**	**2010**	**2004**	**2010**	**2004**	**2010**	**2004**	**2010**
Continental												
Belgium **(BE)**	35.10	38.99	25.31	19.42	17.76	21.29	2.10	0.72	5.17	5.61	14.55	13.96
Germany **(DE)**	32.71	36.69	31.50	24.83	19.16	24.49	1.12	1.69	4.21	3.90	11.31	8.39
France **(FR)**	50.61	56.42	20.86	18.81	15.18	13.74	1.23	0.72	4.60	3.80	7.52	6.51
The Netherlands **(NL)**	18.18	22.55	33.01	23.58	29.67	36.86	1.32	1.29	3.95	5.67	13.88	10.05
Southern												
Spain **(ES)**	39.91	39.05	42.75	34.43	6.58	7.78	0.30	0.92	1.49	3.03	8.97	14.78
Greece **(GR)**	31.75	30.03	41.75	40.04	5.00	5.40	1.38	1.35	2.13	1.69	18.00	21.48
Portugal **(PT)**	52.73	45.21	29.92	26.40	4.46	4.34	0.50	3.80	0.83	3.62	11.57	16.64
Eastern												
The Czech Republic **(CZ)**	60.43	62.09	27.98	28.57	2.02	2.34	0.21	0.69	1.38	1.79	7.98	4.53
Estonia **(EE)**	67.12	56.79	20.40	28.64	4.87	6.46	0.30	0.33	1.37	1.32	5.94	6.46
Poland **(PL)**	56.49	58.58	25.35	26.04	2.59	4.14	0.20	0.20	1.00	1.18	14.37	9.86
Slovenia **(SI)**	45.61	49.60	31.84	30.24	7.46	8.80	0.83	0.48	3.48	3.04	10.78	7.84
Slovakia **(SK)**	66.47	63.62	19.08	18.30	2.60	3.83	0.00	0.21	1.73	1.06	10.12	12.98
The Czech Republic **(CZ)**	55.64	38.57	27.92	38.57	2.38	1.35	0.59	0.00	3.17	1.79	10.30	19.73
Anglo-Saxon												
Great Britain **(GB)**	33.53	36.34	24.51	20.68	24.06	25.44	1.05	2.51	4.51	4.64	12.33	10.40
Ireland **(IE)**	27.86	24.24	37.60	31.65	18.91	15.53	1.36	2.59	3.28	4.00	10.99	22.00
Nordic												
Denmark **(DK)**	66.47	68.31	15.86	13.22	7.85	8.28	0.60	0.80	2.87	4.62	6.34	4.78
Finland **(FI)**	67.72	62.61	19.55	20.77	4.54	5.64	0.50	0.59	2.77	3.71	4.92	6.68
Norway **(NO)**	51.13	55.79	18.24	15.62	19.07	15.20	0.60	1.12	5.01	5.72	5.96	6.56
Denmark **(DK)**	65.93	70.57	14.59	13.38	9.33	9.20	0.70	0.33	3.03	1.84	6.42	4.68
**Average Total**	**47.37**	**46.47**	**27.11**	**24.96**	**11.58**	**13.04**	**0.83**	**1.19**	**3.05**	**3.43**	**10.05**	**10.92**

Notes: Greyed cells show a more unequal gender model compared to the total average, 1: both partners work full time, 2: the man works full time, the woman is non-employed, 3: the man works full time, the woman part time, 4: both work part-time, 5: the woman works more than the man, i.e., the man is non-employed while the woman works part time or the man works part time and the woman full time, 6: both are non-employed or only the man works part time, *: Part-time work is defined as working 1-29 hours per week.

Table A3. Odds ratios estimations for control variables for both genders (Model 1a)

	MALE			FEMALE		
	Exp (b)		Robust S.E	Exp (b)		Robust S.E
Years of education	1.00		[0.012]	0.97	**	[0.013]
Job quality variables						
Job discretion, autonomy and flexibility (0-4 scale: lowest to highest)		**			*	
1	1.01		[0.180]	1.10		[0.119]
2	1.02		[0.095]	1.19		[0.177]
3	1.28		[0.141]	1.45		[0.192]
4	1.16		[0.111]	1.66		[0.372]
Security of Employment (0: Low security, 1: High security)	1.43	***	[0.089]	0.97		[0.052]
Workplace relationships and work motivation (0-4 scale: lowest to highest)		***				
1	1.77		[0.557]	1.10		[0.078]
2	1.24		[0.305]	1.11		[0.074]
3	1.23		[0.246]	1.07		[0.149]
4	1.57		[0.404]	1.29		[0.226]
Safety in employment (0: Low safety, 1: High safety)	2.13	***	[0.159]	2.91	***	[0.550]
Job Intensification (0-3 scale: highest to lowest)		***			***	
1	1.34		[0.058]	1.43		[0.158]
2	1.96		[0.195]	3.06		[0.678]
3	1.98		[0.380]	2.40		[0.487]
Age	1.02	***	[0.005]	1.01	***	[0.003]
Hours worked overtime included	0.91	***	[0.004]	0.93	***	[0.008]
Industry (Ref: Accommodation and food)	***	***		***	***	
Agriculture/Fishing	2.33		[0.585]	1.35		[0.423]
Mining	2.14		[0.683]	4.14		[4.683]
Manufacturing	3.08		[0.638]	2.68		[0.670]
Energy	4.18		[2.530]	4.99		[4.971]
Water Supply Waste	3.46		[1.756]	1.40		[1.007]
Construction	4.82		[0.930]	5.43		[2.125]
Retail	2.94		[0.695]	1.18		[0.314]
Transportation and Storage	1.50		[0.308]	1.54		[0.480]
Infor/Communication	2.27		[0.857]	2.78		[0.659]
Finance	4.41		[0.936]	2.91		[1.310]
Real Estate	6.42		[2.707]	2.76		[1.812]
Prof/Sci/Tech Activities	2.46		[0.515]	3.22		[1.045]
Admin/Support/Serv & Public/Defence/Social Sec	2.37		[0.551]	4.53		[0.968]
Education	3.03		[0.849]	2.02		[0.405]
Health and Social Work	1.67		[0.389]	1.21		[0.268]
Arts, Entertainment and Recreational activities	1.44		[0.819]	2.03		[1.041]
Other Services	3.40		[1.072]	1.65		[0.769]
Occupational category (Ref: Expert mangers)	***	***		***	***	
Expert supervisors	1.61		[0.457]	2.64		[0.839]
Experts	1.63		[0.596]	3.52		[0.978]
Skilled managers	1.17		[0.176]	3.33		[0.956]
Skilled supervisors	0.87		[0.092]	1.75		[0.349]
Skilled workers	1.28		[0.240]	2.58		[0.644]
Low skilled managers	1.05		[0.178]	2.01		[0.447]
Low skilled supervisors	0.81		[0.139]	3.07		[1.034]
Low skilled workers	1.13		[0.110]	3.66		[0.954]
Trade Union Membership (Ref: No)	**	***		**		
Yes currently	0.79		[0.039]	0.76		[0.177]
Yes previously	1.03		[0.081]	0.77		[0.180]
Hampered by health problem (Ref:No)		**			*	
Yes, to some extent	0.99		[0.164]	0.80		[0.135]
Yes, a lot	0.63		[0.107]	0.64		[0.189]
Having dependent children living in the household	1.19	***	[0.074]	1.20	*	[0.118]
Women should be prepared to cut down on paid work for sake of family (Ref: Agree strongly)						
Agree	1.18		[0.129]	1.17		[0.165]
Neither agree nor disagree	1.16		[0.159]	1.31		[0.201]
Disagree	1.09		[0.147]	1.24		[0.210]
Disagree strongly	1.28		[0.173]	1.27		[0.210]
Men should have more right to job than women when jobs are scarce (Ref: Agree strongly)		*			***	
Agree	1.44		[0.363]	1.07		[0.307]

Table A3:Odds ratios estimations for control variables for both genders (Model 1a) (Cont.)

Neither agree nor disagree	1.32		[0.332]	0.74		[0.164]
Disagree	1.40		[0.307]	0.73		[0.168]
Disagree strongly	1.14		[0.285]	0.60		[0.152]
Respondent's partner is working	1.01		[0.109]	0.92		[0.097]
Partner has a higher degree	0.70	***	[0.043]	1.24	**	[0.111]
Satisfied with life in general	1.42	***	[0.084]	2.02	***	[0.280]

Notes: * P<0.10,**P<0.05, ***P<0.01 - No obs: Not enough observation exist in this occupational category for women for estimations to be performed

Table A4. Odds ratios estimations for control variables for both genders (Model 2a)

	MALE			FEMALE		
	Exp (b)		Robust S.E	Exp (b)		Robust S.E
Job quality variables		**			*	
Job discretion, autonomy and flexibility (0-4 scale: lowest to highest)						
1	0.98		[0.295]	0.98		[0.109]
2	1.09		[0.315]	1.29		[0.284]
3	1.08		[0.259]	1.74		[0.466]
4	0.88		[0.173]	1.83		[0.657]
Security of Employment (0: Low security, 1: High security)	1.18	***	[0.097]	1.15		[0.270]
Workplace relationships and work motivation (0-4 scale: lowest to highest)		**			**	
1	1.90		[0.520]	0.81		[0.097]
2	1.49		[0.411]	1.02		[0.090]
3	1.41		[0.208]	0.81		[0.215]
4	1.64		[0.286]	0.81		[0.221]
Safety in employment (0: Low safety, 1: High safety)	2.18	***	[0.200]	2.67	***	[0.317]
Job Intensification (0-3 scale: highest to lowest)		***			***	
1	1.43		[0.225]	1.54		[0.245]
2	2.46		[0.234]	4.14		[0.450]
3	1.64		[0.348]	2.19		[0.468]
Age	1.02	***	[0.007]	1.01	**	[0.007]
Hours worked overtime included	0.90	***	[0.011]	0.93	***	[0.013]
Industry (Ref: Accommodation and food)		***			***	
Agriculture/Fishing	2.09		[1.468]	1.91		[1.002]
Mining	1.75		[0.886]	1.00		[No obs]
Manufacturing	1.48		[0.644]	5.13		[1.285]
Energy	1.42		[1.122]	1.00		[No obs]
Water Supply Waste	1.23		[1.081]	5.36		[5.183]
Construction	2.22		[0.884]	4.35		[3.123]
Retail	2.15		[1.088]	1.81		[0.653]
Transportation and Storage	0.95		[0.442]	1.72		[1.004]
Infor/Communication	0.66		[0.378]	4.53		[1.828]
Finance	1.77		[1.213]	5.70		[2.525]
Real Estate	2.86		[2.866]	1.71		[0.982]
Prof/Sci/Tech Activities	0.95		[0.413]	4.14		[1.364]
Admin/Support/Serv & Public/Defence/Social Sec	1.52		[0.643]	18.67		[5.833]
Education	2.85		[1.427]	3.50		[1.136]
Health and Social Work	1.01		[0.462]	1.68		[0.368]
Arts, Entertainment and Recreational activities	0.52		[0.377]	2.85		[1.423]
Other Services	1.15		[0.452]	3.00		[1.766]
Occupational category (Ref: Expert mangers)		***			***	
Expert supervisors	1.04		[0.435]	1.97		[1.073]
Experts	0.95		[0.304]	2.09		[0.809]
Skilled managers	0.67		[0.114]	1.90		[0.599]
Skilled supervisors	0.50		[0.114]	0.89		[0.114]
Skilled workers	0.63		[0.206]	1.85		[0.396]
Low skilled managers	0.52		[0.264]	1.08		[0.408]
Low skilled supervisors	0.43		[0.093]	2.28		[0.820]
Low skilled workers	0.61		[0.143]	2.98		[0.830]
Trade Union Membership (Ref: No)		***			***	
Yes currently	0.70		[0.063]	0.64		[0.104]
Yes previously	0.90		[0.068]	1.04		[0.210]
Hampered by health problem (Ref: No)		***				
Yes, to some extent	0.95		[0.202]	0.92		[0.206]
Yes, a lot	0.41		[0.170]	1.01		[0.719]
Having dependent children living in the household	1.09		[0.063]	1.26	**	[0.137]
Women should be prepared to cut down on paid work for sake of family (Ref: Agree Strongly)					***	
Agree	1.23		[0.317]	1.23		[0.273]
Neither agree nor disagree	1.13		[0.299]	1.17		[0.481]
Disagree	1.34		[0.288]	1.46		[0.534]
Disagree strongly	1.44		[0.361]	1.40		[0.604]
Men should have more right to job than women when jobs are scarce (Ref: Agree Strongly)		***			***	
Agree	1.46		[0.455]	1.30		[0.375]
Neither agree nor disagree	1.39		[0.344]	0.92		[0.258]

Table A4: Odds ratios estimations for control variables for both genders (Model 2a) (Cont.)

Disagree	1.34		[0.342]	0.87		[0.305]
Disagree strongly	1.03		[0.245]	0.70		[0.209]
Respondent's partner is working	1.10		[0.140]	0.63	*	[0.171]
Partner has a higher degree	0.56	***	[0.060]	1.27		[0.254]
Total time people in home spend on housework, hours typical weekday	0.93	***	[0.019]	0.98		[0.017]
Part your partner spend of total time housework on typical weekday (Ref: Not at all)		***			***	
Up to 1/4 of the time	2.58		[1.156]	1.23		[0.273]
More than 1/4, up to 1/2 of time	2.18		[0.899]	0.87		[0.134]
More than 1/2, up to 3/4 of time	1.77		[1.028]	0.64		[0.248]
More than 3/4, less than all of time	2.33		[1.234]	1.49		[0.785]
All or nearly all of time	1.51		[0.740]	1.21		[0.664]
Satisfied with life in general	1.35	**	[0.176]	1.82	***	[0.247]

Notes:

* p<0.10,**p<0.05, ***p<0.01

No obs: Not enough observation exist in this occupational category for women for estimations to be performed

Table A5. Odds ratios estimations for control variables for both genders (Model 3a)

	MALE			FEMALE		
	Exp (b)		Robust S.E	Exp (b)		Robust S.E
Job quality variables						
Job discretion, autonomy and flexibility (0-4 scale: lowest to highest,)						
1	1.01	***	[0.213]	1.31		[0.293]
2	0.92		[0.174]	1.16		[0.167]
3	1.44		[0.111]	1.29		[0.158]
4	1.41		[0.149]	1.56		[0.449]
Security of Employment (0: Low security, 1: High security)	1.69	***	[0.231]	0.88		[0.167]
Workplace relationships and work motivation (0-4 scale: lowest to highest)		***				
1	1.85		[0.758]	1.31		[0.208]
2	1.20		[0.332]	1.17		[0.206]
3	1.20		[0.304]	1.34		[0.337]
4	1.59		[0.449]	1.65		[0.524]
Safety in employment (0: Low safety, 1: High safety)	1.93		[0.156]	3.51	***	[0.915]
Job Intensification (0-3 scale: highest to lowest)		***			***	
1	1.46	***	[0.146]	1.38	***	[0.258]
2	1.94		[0.164]	2.50		[1.067]
3	3.64		[1.213]	2.67		[0.627]
Age	1.02	***	[0.006]	1.01	***	[0.004]
Hours worked overtime included	0.92	***	[0.146]	0.93	***	[0.258]
Industry (Ref: Accommodation and food)		***			***	
Agriculture/Fishing	2.36		[0.937]	0.83		[0.302]
Mining	2.58		[1.328]	2.00		[2.720]
Manufacturing	5.31		[1.873]	1.66		[0.736]
Energy	12.7		[10.169]	1.80		[1.748]
Water Supply Waste	9.18		[4.375]	0.43		[0.325]
Construction	8.60		[2.014]	9.51		[3.072]
Retail	4.00		[1.535]	0.91		[0.300]
Transportation and Storage	2.03		[0.638]	1.47		[0.764]
Infor/Communication	6.67		[2.214]	2.74		[1.515]
Finance	9.63		[2.597]	1.71		[1.082]
Real Estate	24.4		[22.475]	1.00		[No obs]
Prof/Sci/Tech Activities	5.59		[1.505]	3.48		[1.664]
Admin/Support/Serv & Public/Defence/Social Sec	3.49		[1.218]	2.27		[0.782]
Education	2.94		[1.015]	1.40		[0.341]
Health and Social Work	2.11		[0.716]	0.96		[0.321]
Arts, Entertainment and Recreational activities	5.45		[4.582]	1.54		[1.123]
Other Services	8.40		[3.288]	1.14		[0.686]
Occupational category (Ref: Expert mangers)		***			***	
Expert supervisors	2.44		[1.07]	3.08		[1.368]
Experts	2.66		[1.126]	5.16		[1.514]
Skilled managers	2.13		[0.53]	5.88		[3.114]
Skilled supervisors	1.55		[0.246]	2.52		[0.884]
Skilled workers	2.61		[0.616]	3.21		[1.118]
Low skilled managers	1.99		[0.5]	2.69		[1.026]
Low skilled supervisors	1.39		[0.244]	3.46		[1.457]
Low skilled workers	2.14		[0.525]	3.99		[1.300]
Trade Union Membership (Ref: No)					**	
Yes currently	0.86		[0.124]	0.82		[0.262]
Yes previously	1.20		[0.203]	0.53		[0.175]
Hampered by health problem (Ref: No)						
Yes, to some extent	1.09		[0.18]	0.83		[0.173]
Yes, a lot	0.89		[0.579]	0.61		[0.279]
Having dependent children living in the household	1.33	**	[0.152]	1.18	*	[0.115]
Women should be prepared to cut down on paid work for sake of family (Ref: Agree Strongly)					**	
Agree	1.24		[0.277]	0.95		[0.152]
Neither agree nor disagree	1.12		[0.206]	1.42		[0.338]
Disagree	0.89		[0.191]	1.03		[0.156]
Disagree strongly	1.25		[0.303]	1.02		[0.273]
Men should have more right to job than women when jobs are scarce (Ref: Agree Strongly)		***			*	
Agree	2.20		[0.962]	0.59		[0.298]
Neither agree nor disagree	1.76		[0.828]	0.48		[0.148]

Table A5: Odds ratios estimations for control variables for both genders (Model 3a) (Cont.)

Disagree	2.08		[1.028]	0.54		[0.136]
Disagree strongly	1.62		[0.802]	0.44		[0.155]
Respondent's partner is working	1.28		[0.244]	1.23		[0.24]
Partner has a higher degree	0.70	***	[0.076]	1.20	**	[0.109]
Total time people in home spend on housework, hours typical weekday	1.03	**	[0.013]	1.00		[0.006]
Total hours per week your partner spends in housework	0.99		[0.008]	0.98	***	[0.005]
Satisfied with life in general	1.51	***	[0.191]	2.46	***	[0.643]

Notes:* p<0.10,**p<0.05, ***p<0.01

No obs: Not enough observation exist in this occupational category for women for estimations to be performed

Table A6: Bivariate linear regressions (Second Step)

	Difference 2004-2010 (Model 1b)				2004 (Model 2b)				2010 (Model 3b)			
	Male DWLB		Female DWLB		Male ($MWLB_{ed}$)		Female ($FWLB_{ed}$)		Male ($MWLB_{ed}$)		Female ($FWLB_{ed}$)	
	Coef.	S.E	Coef.	S.E	Coef.	S.E	Coef.	S.E	Coef.	S.E	Coef.	S.E
**GDP Growth rate**	-0.065	0.580	**0.104***	**0.049**	-0.015	0.009	**0.025****	**0.009**	0.011	0.010	**-0.018***	**0.010**
R^2	0.08		**0.23**		0.15		**0.34**		0.08		**0.18**	
**Unemployment rate**	-0.022	0.361	-0.003	0.034	**-0.01*****	**0.043**	-0.0002	0.004	0.002	0.006	-0.0003	0.006
R^2	0.01		0.0003		**0.38**		0.0002		0.004		0.0001	
**Economy's state (subjective measurement) 2004**					0.005	0.012	0.017	0.0120				
R^2					0.13		0.11					
**Economy's state (subjective measurement) 2010**	-010	0.062	**0.102***	**0.517**					0.007	0.011	0.0010	0.112
R^2	0.001		**0.20**						0.03		0.0006	
**GDP Growth rate 2004-2010 difference**	-0.045	0.037	**0.066***	**0.312**					-0.002	0.007	**-0.012***	**0.006**
R^2	0.09		**0.22**						0.008		**0.19**	
**Unemployment rate 2004-2010 difference**	0.0234	0.276	**-0.052****	**0.0224**					-0.007	0.005	**0.009***	**0.004**
R^2	0.05		**0.26**						0.14		**0.22**	
**Economy's state (subjective measurement) 2004-2010 difference**	-0.058	0.625	0.070	0.565					0.011	0.011	-0.012	0.110
R^2	0.05		0.09						0.06		0.08	

Notes:* $p<0.10$,** $p<0.05$, *** $p<0.01$

Note: Significant relationships are in bold fonds
